# Disintegration of the Second‐Generation Precice Bone Transport Nail During Removal Is Still an Issue: A Case Report

**DOI:** 10.1155/cro/5629022

**Published:** 2026-03-25

**Authors:** Rikke Gravesen, Daniel Wæver, Juozas Petruskevicius, Jan Duedal Rölfing

**Affiliations:** ^1^ Department of Orthopaedics and Traumatology, Aarhus University Hospital, Aarhus, Denmark, auh.dk; ^2^ Department of Orthopaedic Surgery, Regional Hospital Randers, Randers, Denmark, regionshospitalet-randers.dk

**Keywords:** bone lengthening, bone transport nail, hardware removal, intraoperative complications, Precice

## Abstract

**Background:**

Intramedullary bone transport nails (BTNs) have become an increasingly common method for managing bone defects. The Precice BTN (Globus Medical, Inc.) was previously associated with mechanical failures including intramedullary disintegration during elective nail removal. These issues were reportedly resolved in the second generation BTN.

**Results:**

Adhering to the CARE checklist, we describe a case involving a 23‐year‐old male with a posttraumatic bone defect following a motorcycle accident. He sustained a Gustilo–Anderson Type IIIB comminuted right fracture with a 5 cm bone defect. The leg was initially stabilized with external fixation. After several debridements, the fracture was internally stabilized using a regular trauma nail while the soft‐tissue defect was covered with a free flap and split‐thickness skin grafting. Subsequently, the 5 cm bone defect was managed using a second‐generation BTN. After 8 months, the patient underwent a planned nail removal according to the manufacturer′s instructions. Just as with the first‐generation BTN, the nail disintegrated within the medullary canal during the procedure and separated into multiple components. These internal components were cumbersomely removed using long forceps.

**Conclusion:**

Mechanical failure, specifically intramedullary disintegration, still occurs with the second‐generation BTN upon nail removal. The retention plug of the first‐generation BTN should thus be used despite instructions from the manufacturer that the problem has been solved in the current generation BTN. Appropriate instruments to manage potential nail disintegration should be present. The case underlines the need for further design improvements in future generations of BTN.

## 1. Background

Bone transport and lengthening using an externally applied fixator is a well‐established treatment for limb length discrepancies; however, it carries a high risk of complications such as infection, pain, and immobility [1, 2]. To mitigate these risks and improve patient comfort, internal techniques—such as intramedullary bone transport nails (BTNs)—have been introduced [3–7].

The Precice BTN from Globus Medical, Inc. (formerly Nuvasive Specialized Orthopedics, Inc.) is designed for all internal bone transport. Gradual bone transport is magnetically induced with an external remote controller. The system is indicated for patients over 18 years of age with segmental defects up to 10 cm caused by trauma or tumors [8]. The manufacturer recommends removing the device within 1 year of implantation [9]. A systematic review has shown that device‐related issues are among the most common complications associated with intramedullary nailing [5].

The magnetically driven BTN is made out of Biodur 108, and the material itself is prone to corrosion [10]. Moreover, the first‐generation Precice Biodur intramedullary devices occasionally fractured or disintegrated during removal [11–13]. Consequently, the manufacturer developed and recommended the use of a retention plug. However, the plug should not be needed for the currently available second‐generation BTN.

## 2. Case Report

### 2.1. Patient Information and Clinical Findings

A 23‐year‐old previously healthy male presented to the emergency department (ED) after a motorcycle accident. He had sustained an open fracture of his lower right leg (Gustilo–Anderson IIIB), and distal pulses were initially absent (Figure 1a).

Figure 1AP view of the right lower leg. High‐energy fracture before (a) and after external (b) and internal (c) temporary fixation resulting in a 5 cm bone defect.

**Figure:**
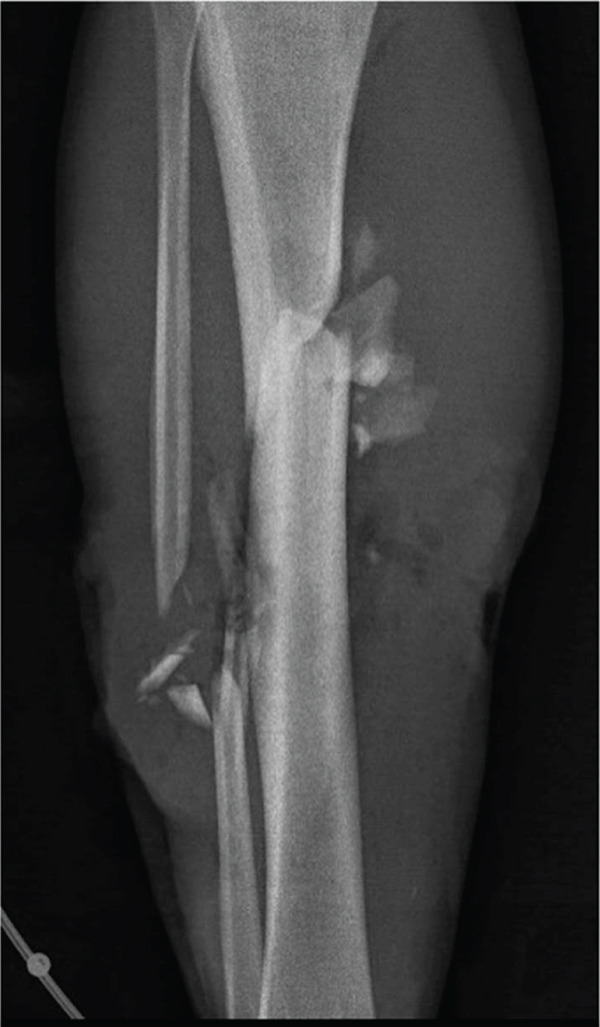
(a)

**Figure:**
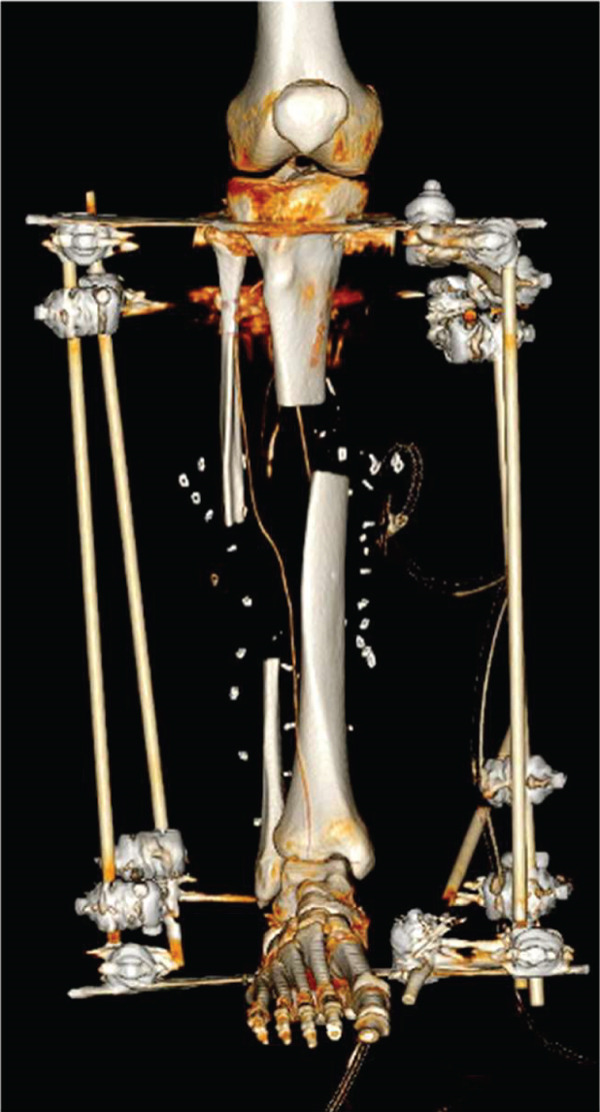
(b)

**Figure:**
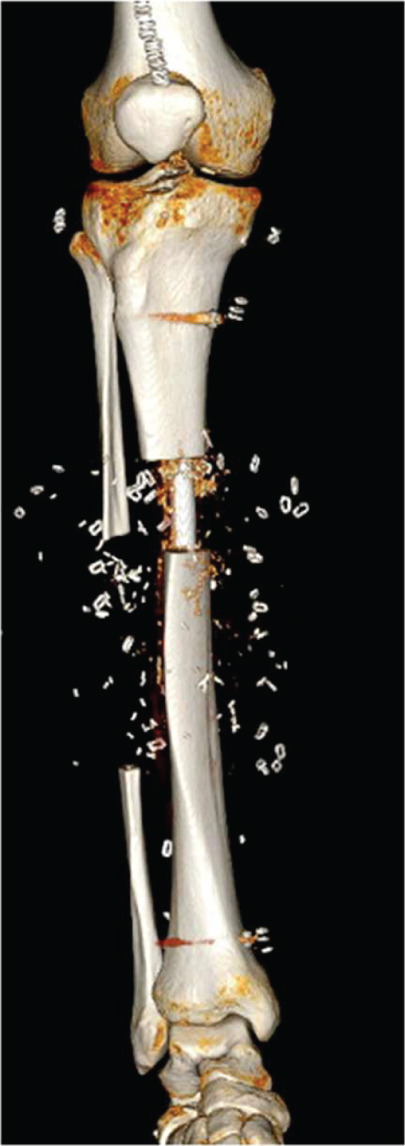
(c)

CT angiography of the lower extremity revealed the comminuted fracture as well as suspicion of a minor arterial injury. The scan was repeated 4 days later and showed restored blood flow through the artery. The CT scan also showed a bone defect of approximately 45 mm, a fibular bone defect of 110 mm, and a leg length discrepancy of 5 mm (Figure 1b).

### 2.2. Therapeutic Intervention

**Figure 2:**
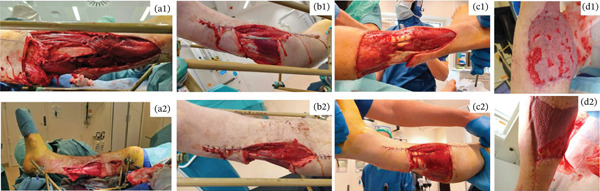
Top row (a1–d1): Lateral side of the right lower leg. Bottom row (a2–d2): Medial side of the right lower leg. (a) Second look of the wounds. (b) Third wound revision of the skin and muscle defects on the lower right leg. (c) Wounds after numerous wound revision and 9 days of using VAC. (d) Split‐thickness skin graft.

Initially, the lower leg was stabilized using monolateral external fixation (Figure 1b). After several debridements using vacuum‐assisted closure (VAC) and split‐thickness skin grafting (Figure 2), the fracture was stabilized via a suprapatellar approach with an intramedullary Stryker T2 Alpha trauma nail (Figure 1c) while rebuilding the existing monolateral frame into a tripod for easy access for plastic surgery and protection of the planned free flap. Once the fracture was internally stabilized, the patient underwent reconstructive surgery with a free latissimus dorsi skin–muscle flap to repair the large skin and muscle defect on the right leg including tunneling of the muscle around the anterior part of the tibia with fragile tissue coverage. (Figure 2d).

After sufficient soft tissue coverage and no signs of infection, it was decided to treat the 45‐mm bone defect and 5‐mm leg length discrepancy using a second‐generation Precice BTN (antegrade tibia, 11.5 × 360 mm, 3 and 7‐cm transport slots (REF: BT115‐10SJ360‐7, LOT: 2060307, expiration date: 2025‐05‐31)) 3 weeks after the flap surgery. The surgery performed in April 2024 proceeded as planned. The osteotomy was performed by modified De Bastiani technique (Figure 3a). Bone transport was monitored with multiple x‐rays over the following months to ensure adequate bone formation (Figures 3b, 3c, and 3d).

Figure 3All internal bone transport with the second‐generation Precice BTN. AP view of the right lower leg (a) postoperatively. (b) Six weeks postoperative radiograph: approx. 2 cm of transport; the distal screws have been removed 3 weeks prior due to infection. (c) Eight weeks postoperatively after implantation with two new screws distally to lock the nail and protect the ankle joint. d) Four months postoperative radiographs with completed transport before initiation of compression of the docking site.

**Figure:**
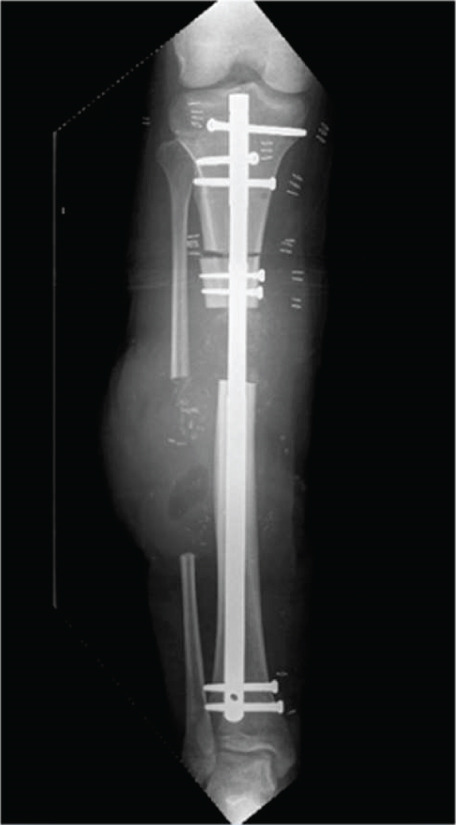
(a)

**Figure:**
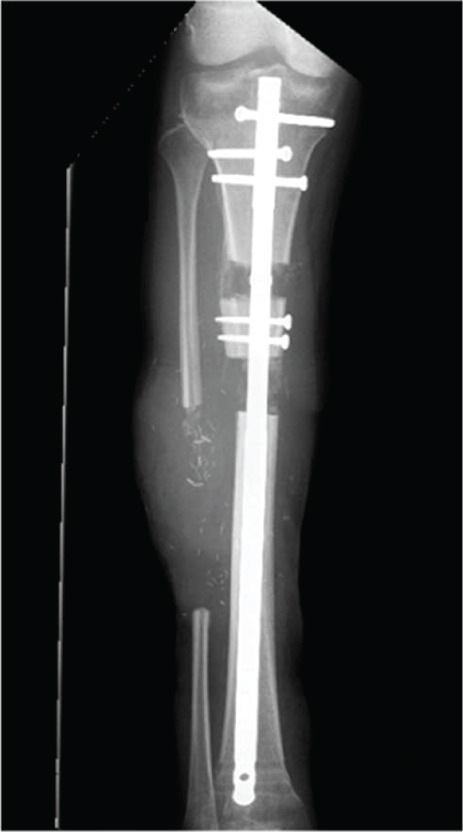
(b)

**Figure:**
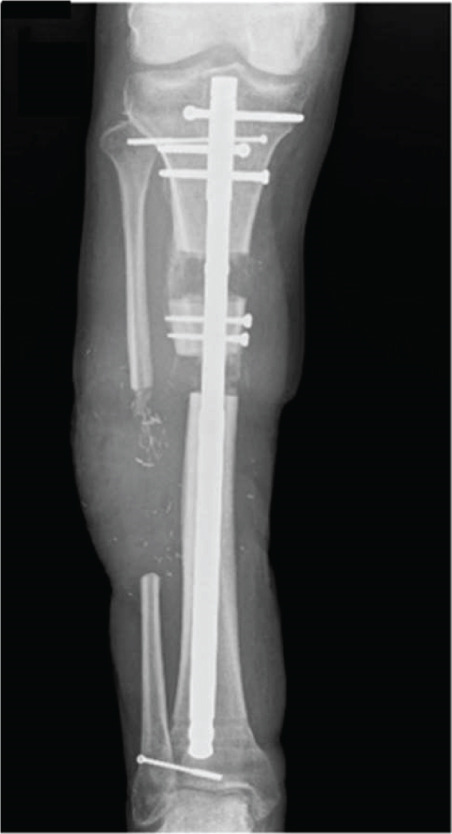
(c)

**Figure:**
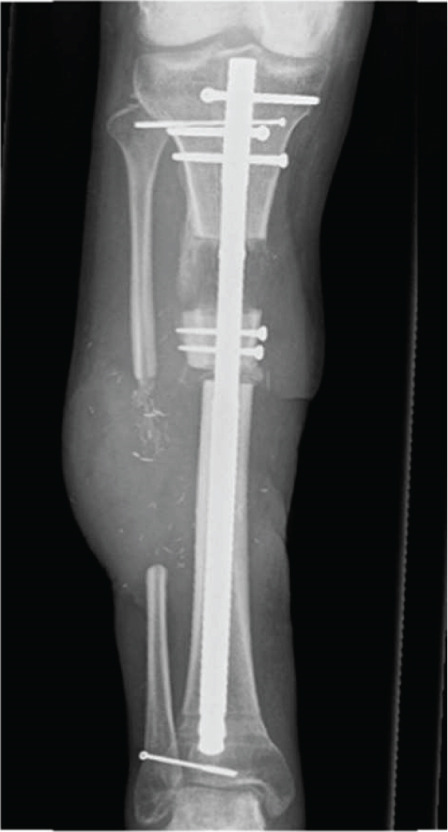
(d)

Postoperatively, the patient participated in a specialized physical therapy and was allowed 20‐kg weight bearing on the right leg along with unloaded movement training. Six weeks after surgery, the patient began swimming, and after three months, he progressed to full weight bearing. During the bone transport period, the patient received paracetamol, morphine, and pregabalin for pain management.

Twenty‐four days after BTN insertion, the patient experienced local pain and redness at the distal locking screws. He was afebrile with a C‐reactive protein (CRP) level of 41 mg/L; however, a deep infection with abscess had developed. X‐rays also showed signs of bone resorption/infection. A debridement, antibiotics, and implant retention (DAIR) procedure was performed. Initial treatment included intravenous antibiotics with both vancomycin and carbapenem, supplementing the debridement and removal of the two distal screws. Tests confirmed a *Staphylococcus aureus* infection, and IV antibiotics were changed to dicloxacillin. Meanwhile, the transport continued. After the infection resolved, an AP locking screw and a tibiofibular screw were inserted to protect the length of the tibia as well as the ankle joint.

### 2.3. Follow‐Up and Outcome

After 8 months and 5 cm bone transport, surgical removal of the BTN was planned, see Table 1.

**Table 1:** Step by step approach of the surgery.

**Step by step description of the removal of the BTN:** 1: Suprapatellar approach. 2: Uncomplicated removal of the endcap and all locking bolts/screws. 3: Removal of the transport nail using backslapping. **The distal part of the transport nail disintegrated, and its internal components remained in the intramedullary cavity.** 4: Using the image intensifier and a long forceps the pieces were removed one by one. 5: It was not possible to retrieve all the pieces with 43‐cm long forceps via the suprapatellar approach. 6: An additional infrapatellar approach was made. 7: The remaining pieces were removed via the infrapatellar approach. 8: A Stryker T2 Alpha Tibial Nail was inserted. **Specifics:** **Blood loss:** 100 mL **Operating time:** 158 min **Fluoroscopy time:** approx. 12 min, that is, excessive; difficult to grasp the individual components, which were pushed back and forth; final check no metal left. **Instruments (used in retrieval):** – 43‐cm cement grasping forceps**—**Waldemar Link GmbH, Hamburg, Germany, Ref. No. 130‐744 – Stryker T2 Alpha nail **Instruments that should be at site:** – Order + use the dedicated retention plug from the first generation of the nail and insert into distal locking holes after locking bolt removal and before backslapping—this should prevent disintegration. – In case of disintegration: ○ Long forceps/cement removal forceps with good jaws ○ Flexible reamers

Due to personal experiences with disintegration of the first‐generation BTN upon nail removal, the surgeons consulted with the distributor of the nail to clarify if insertion of the retention plug into the distal locking holes was still needed to prevent disintegration of the nail. The distributor confirmed that no retention plug was needed in second‐generation BTN, and that the nail could be safely removed in its entirety.

**Figure 4:**
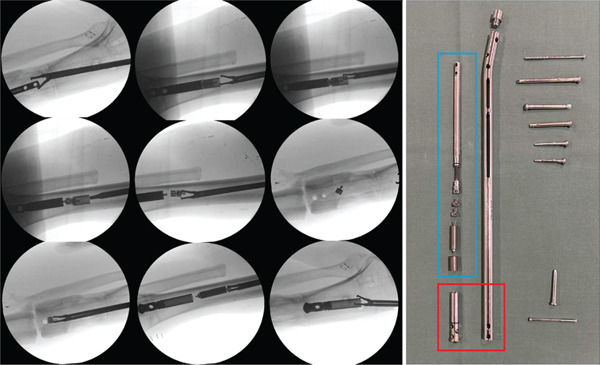
Perioperative x‐rays showing the retrieval of components of the disintegrated second‐generation BTN using a long grasping forceps (43 cm). Red box: point of failure, that is, upon backslapping the distal inner part (on the left) disintegrated from the outer sleeve/nail, which left both the tip and the remaining internal components (blue box) inside the intramedullary cavity.

Through a suprapatellar approach, a Kirschner wire was inserted into the proximal end of the device. The endcap and screws were removed without complications. However, during removal of the transport nail it disconnected intramedullary into multiple components (Figure 4). The pieces had to be removed one by one using a 43‐cm grasping forceps (Figure 4; Waldemar Link GmbH, Hamburg, Germany, Ref. No. 130‐744). Unfortunately, the forceps was not long enough to retrieve the pieces through the suprapatellar approach, and an additional infrapatellar approach had to be made. After complete removal of the pieces of transport nail, a Stryker T2 Alpha nail was inserted. The disintegration of the nail resulted in significantly longer operating time, an unplanned infrapatellar surgical approach and prolonged use of fluoroscopy to retrieve all components one by one. The surgery lasted 158 min, there was a blood loss of 100 mL, and the fluoroscopy time was 12 min and 1 s.

**Figure 5:**
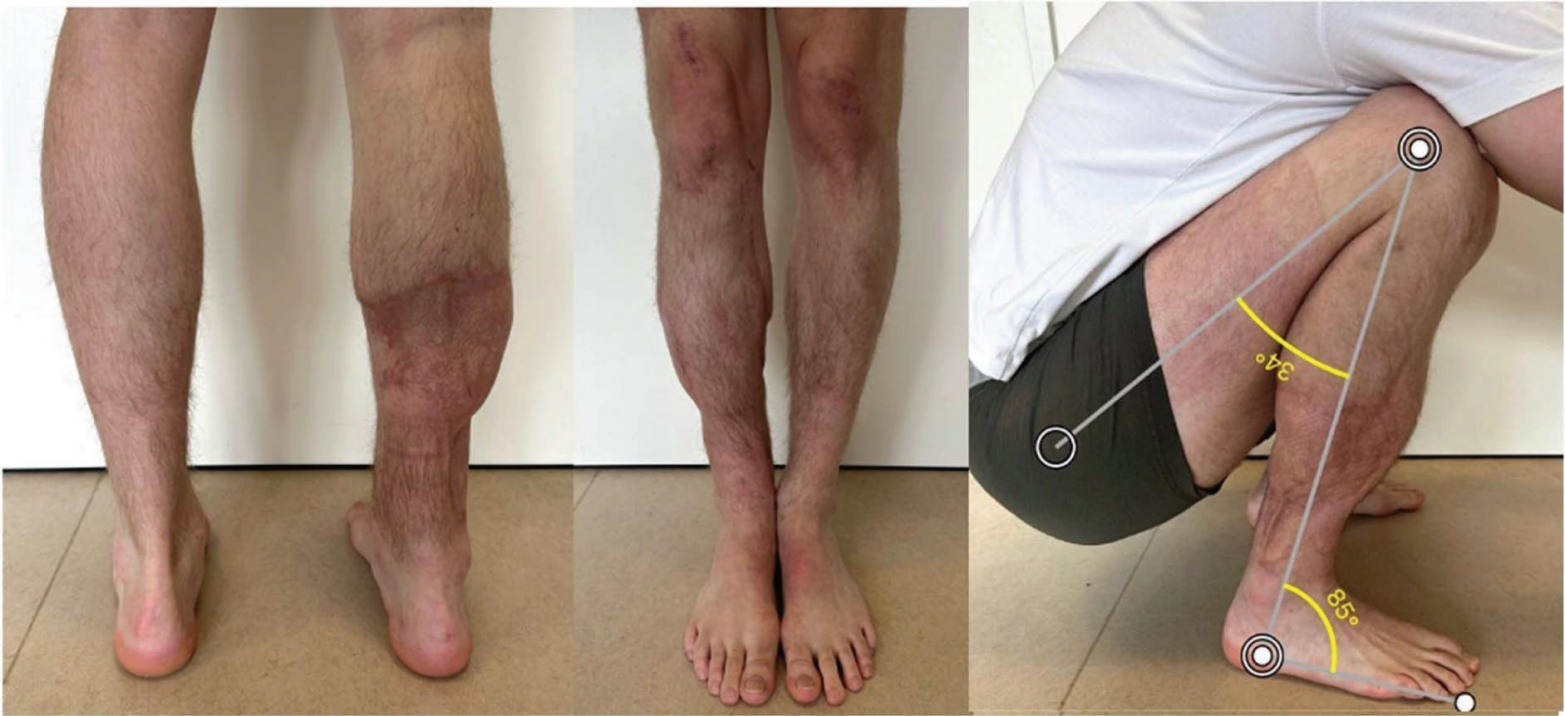
Healed soft tissues, good alignment, and excellent range of motion at the latest follow‐up.

At the latest follow‐up 8 months after removal of the BTN, the patient had no signs of infection and no significant pain. The patient had returned to over fulltime labor as kitchen staff and waiter. Active range of motion (ROM) of the right knee was 10°–146°, passive ROM of 0°–146°. ROM of the right ankle with extended knee: 35° of plantarflexion and 5° of dorsiflexion (Figure 5). He did not use any assistive devices and could fully weight‐bear with a minor limp, but still has reduced muscle strength and endurance.

## 3. Discussion

Disintegration of the distal part of the first‐generation BTN has been well‐established. Consequently, the manufacturer had developed a retention plug, which should be inserted instead of a locking screw in the distal part of the nail [14, 15]. This plug has the same length as the outer diameter of the nail and thus keeps the inner sleeve of the distal portion of the nail attached to the body of the nail. The local distributor reconfirming with the manufacturer stated that this plug is no longer needed in the second‐generation BTN. Our case clearly documents that the second generation of the Precice BTN still disintegrates, and the continued use of the retention plug is thus still needed. The manufacturer has since been made aware of the incidence.

Although all‐internal bone transport holds many advantages over external options, our case report underlines some challenges, that is, deep infection necessitating two additional surgeries, DAIR and secondary insertion of new locking screws. This is likely partially due to the prolonged time without soft tissue coverage, that is, 11 days before definite soft tissue management free flap. Moreover, due to the motor, implant removal is required according to the instructions for use and may be more complicated than anticipated. We assess that the disintegration of the BTN was purely mechanical in nature and was not related to the infection or to the subsequent surgeries. Surgeons and patients should be aware of these risks. Although the disintegration and corrosion were observed in Precice BTN made out of Biodur 108, complications are also seen in bone transport cases with BTN from other manufactures [5].

Halvorson et al. presented a case of a 34‐year‐old man with an intra‐articular nonunion in the tibia following a fracture. Similar to our case, the patient was treated with a Precice BTN and bone transport over 3 months, with planned removal of the nail after 2 years—1 year later than recommended by the manufacturer. During removal, the transport nail disintegrated when it was extracted by backslapping, causing the nail to disconnect into multiple parts. The Stryde BTN has been recalled since February 2021 [14]. Also in 2024, a case report regarding the removal of a broken BTN emerged. Here, the internal components break prior to planned removal, and the disintegration of the nail was thus not preventable as it was in our case [11]. The manufacture released a “Field Safety Notice” on July 27, 2020 and released a retention plug to prevent this issue [16]. The use of the retention plug is described and illustrated in detail by Quinnan in 2021, in brief the distal locking bolts are exchanged for a short *retention plug*, which has the exact same diameter as the outer nail, that is keeping the internal tip of the nail and the outer sleave connected [17].

## 4. Conclusion

Physicians must be aware of the risk of intramedullary nail disintegration during planned removal of the device. The current generation of the BTN still carries this problem, and the retention plug should be used. Moreover, the authors recommend having a retention plug and instruments for disintegrated nails on site in cases of urgent nail removal, for example, due to deep infection. This case underlines the need for further design improvements in future generations of BTN.

## Funding

No funding was received for this manuscript.

## Disclosure

The abstract of this case report was presented as a poster at the 2025 Congress of the Danish Orthopaedic Society (DOS) and published in the DOS Abstract Book 2025[18].

## Consent

Written and verbal consent from the patient was obtained by the co‐author.

## Conflicts of Interest

J.D.R. is giving paid lectures and workshops for Orthofix Srl., Bussolengo, Italy. The remaining authors declare no conflicts of interest.

## Supporting information


**Supporting Information** Additional supporting information can be found online in the Supporting Information section. The case report was written in accordance with the CARE checklist, which has been submitted as supporting information.

## Data Availability

Data sharing is not applicable to this article as no datasets were generated or analyzed during the current study.
