# Supplementary material for: Disintegration of the Second‐Generation Precice Bone Transport Nail During Removal Is Still an Issue: A Case Report
Source: Case Rep Orthop. 2026 Mar 25;2026:5629022. doi: 10.1155/cro/5629022 (PMC13150965; doi:10.1155/cro/5629022)
Supplement: Supplementary file 1 — Supporting Information Additional supporting information can be found online in the Supporting Information section. The case report was written in accordance with the CARE checklist, which has been submitted as supporting information. [file CRO-2026-5629022-s001.pdf]

CARE
case report guidelines

CARE Checklist of information to include when writing a case report

(cc) BY-NC-ND

Topic	Item	Checklist item description	Reported on Line
Title	1	The diagnosis or intervention of primary focus followed by the words "case report"	Page 1, Line 2
Key Words	2	2 to 5 key words that identify diagnoses or interventions in this case report, including "case report"	Page 2, Last 2 lines
Abstract (no references)	3a	Introduction: What is unique about this case and what does it add to the scientific literature?	Page 2, Line 16
	3b	Main symptoms and/or important clinical findings	Page 2, Line 6-8
	3c	The main diagnoses, therapeutic interventions, and outcomes	Page 2, Line 8-15
	3d	Conclusion—What is the main "take-away" lesson(s) from this case?	Page 2, line 16-20
Introduction	4	One or two paragraphs summarizing why this case is unique (**may include references**)	Page 9, line 7-8
Patient Information	5a	De-identified patient specific information.	Page 3, Line 18
	5b	Primary concerns and symptoms of the patient.	Page 3, Line 19-22
	5c	Medical, family, and psycho-social history including relevant genetic information	Page 3, line 18
	5d	Relevant past interventions with outcomes	%
Clinical Findings	6	Describe significant physical examination (PE) and important clinical findings.	Page 3, line 19-22 + page 4 line1-2
Timeline	7	Historical and current information from this episode of care organized as a timeline	%
Diagnostic Assessment	8a	Diagnostic testing (such as PE, laboratory testing, imaging, surveys).	Page 3, line 21-22 + page 4, line 1-2
	8b	Diagnostic challenges (such as access to testing, financial, or cultural)	Page 3, line 21-22
	8c	Diagnosis (including other diagnoses considered)	Page 4, line 1-2
	8d	Prognosis (such as staging in oncology) where applicable	%
Therapeutic Intervention	9a	Types of therapeutic intervention (such as pharmacologic, surgical, preventive, self-care)	Page 4, Line 3- Page
	9b	Administration of therapeutic intervention (such as dosage, strength, duration)	%
	9c	Changes in therapeutic intervention (with rationale)	page 5, line 1
Follow-up and Outcomes	10a	Clinician and patient-assessed outcomes (if available)	page 8, line 6-111
	10b	Important follow-up diagnostic and other test results	page 5, line 8-13
	10c	Intervention adherence and tolerability (How was this assessed?)	page 6, line 1-6
	10d	Adverse and unanticipated events	Page6, line 6-21
Discussion	11a	A scientific discussion of the strengths AND limitations associated with this case report	Page 9, line 7
	11b	Discussion of the relevant medical literature **with references**.	page 9, line 1 + page 10, line 5
	11c	The scientific rationale for any conclusions (including assessment of possible causes)	page 10, line 18
	11d	The primary "take-away" lessons of this case report (without references) in a one paragraph conclusion	page 10, line 18
Patient Perspective	12	The patient should share their perspective in one to two paragraphs on the treatment(s) they received	%
Informed Consent	13	Did the patient give informed consent? Please provide if requested	Yes [x] No [ ]
